# Editorial

**Published:** 1844-01-13

**Authors:** 


### THE MEDICAL EXAMINER.

PHILADELPHIA, JANUARY 13, 1844.

The professional engagements of the late Editor of this Journal, oblige him to relinquish his connection with it; a connection which, with a short intermission, has existed since the establishment of the Examiner, a period of six years. He trusts that the same patronage which has heretofore been so liberally extended to the Journal, will be continued to it under his successor.

---

In entering upon the duties of his new vocation, the Editor feels constrained to avoid all lengthened promises and exorbitant claims to professional confidence and support. The task has fallen upon him suddenly and unexpectedly; in the little time which has elapsed, however, he has succeeded in making such arrangements as will, he trusts, enable him to render the pages of the Examiner useful and interesting to all who may favour it with their patronage. The prospectus, issued by his predecessor, for the present year, is republished, slightly modified, on the cover of the present number, and to that he refers for information in regard to the general plan of the work; to what is therein stated, he now merely adds the expression of his determination to conduct it on broad and liberal principles, and on no account to admit anything to its columns which can be regarded as at all personal or unprofessional.

---

### MEDICAL CLASSES.

Information from all parts of the country shows an unusually large attendance of Students at the various Medical Colleges, the present winter. This may be accounted for by the embarrassed condition of agriculture and commerce during the last few years, which has probably induced more than the usual proportion of young men to look to the learned professions; and partly, perhaps, by the fact that many have been kept back by the general pecuniary distress which has prevailed, who now find means to avail themselves of the advantages of public lectures. At the two Schools in Philadelphia, the number of Students is greater than at any former season. The University of Pennsylvania has more than her average class, and the number at the Jefferson Medical College exceeds that of last year, by nearly one hundred.

### INTRODUCTORY LECTURES.

The present has been a fruitful season of Introductory Lectures. Most if not all the schools of the country, village and metropolitan, have furnished their quota. As space is afforded, we shall notice those we have received, and occasionally take the liberty of making extracts from them, on subjects likely to interest our readers. The only one for which we have room in the present number is from the veteran Professor of Chemistry, in the University of Pennsylvania. It is entitled a "Lecture Introductory to a Course on Chemistry, in the University of Pennsylvania, delivered November 7th, 1843, by Robert Hare."

The following "remarks" will exhibit his opinion of "Liebig's physiological speculations."

"In these I conceive he has in various instances been bold, hasty, inconsiderate, and inaccurate, but still he has advanced many ingenious ideas which are likely to be highly servicable to physiological chemistry. He has quite as much merit in holding up ideas, before existing, in a new and popular form, as in suggesting such as are altogether original. Yet I would liken him to a military leader, who, after marching through a country, with drums beating and colours flying, should have his trumpets loudly sounded, as if a complete conquest had been effected, while leaving behind him many fortresses, of which the knowledge had prevented more cautious and considerate leaders from having previously undertaken the same expedition. Nevertheless, by these means the philosopher of Giessen has excited a degree of attention, in the great mass of physicians and agriculturists, which had never been gained, had he neither deluded himself nor the readers of his essays with the prospect of an elucidation of the mysteries of animal and vegetable physiology, which it is beyond the present state of chemistry to afford. Moreover, the popularity which he has thus gained, may lead others to follow in the same path, who may rectify his errors and remedy some of his omissions without impairing what is really true in his doctrines.

"There can be no better exemplification of the errors to which Liebig is addicted, than his adoption of the following maxim. '*There are many ways to the highest pinnacle of a mountnin, but those only can hope to reach it who keep the summit constantly in view.*' It must be evident to every person of any experience, in ascending mountains, that although it may be necessary to keep the *bearing* of the summit in *mind*, our eyes must be upon the path; and that, in most cases, the safest and easiest mode of access, causes the summit to be concealed for a time. A person who should implicitly follow Liebig's advice, would probably fall over some precipice, or tumble into some fissure which might escape notice while keeping the summit of the mountain *constantly* in view. Is not the fallacious rule of action above quoted, a good figurative illustration of a theorist, who, keeping his mind too much upon some hypothetical acme, overlooks insurmountable objections which a close attention to facts would make evident? Has not Liebig exemplified his own course?" * * * *

"One of the greatest services rendered by the author, whose opinions are under consideration is, as I think, in directing attention to the different offices performed by two classes of vegetable products which may be distinguished as nitrogenized and as devoid of nitrogen. All the various species of sugar, starch, gum, or mucilage, oil, fat, and gelatine, are represented as having a tendency rather to go to the support of the respiratory process, or to produce obesity; while the fibrin and albumen of flesh and blood are sustained by those portions of animal and vegetable food which contain nitrogen in nearly the same proportion as it exists in them. The greater vigour of a horse when fed on oats or maize, is in this way explained, by the greater proportion of matter contained in such grain, which is of a nature to compensate the wear of the muscles. * *

"Highly worthy of consideration, also, are Liebig's suggestions respecting the services rendered by theine, a peculiarly highly nitrogenized principle, common both to tea and coffee. Liebig ingeniously shows that this principle requires only an addition of water and oxygen in order to convert it into taurine, an active principle of the bile. The extensive use of tea and coffee by civilized nations thus appears to have been the result of a sort of instinctive empyrical research, leading to beneficial results, which physicians were heretofore unable to appreciate or explain. In fact, as food, coffee and tea were heretofore considered as almost valueless; but now it appears that they serve to furnish nitrogen in a more concentrated form to those whose indolent habits might be incompatible with the consumption of sufficient quantity of ordinary nutriment to obtain a requisite quantity of that element." * * *

"It seems to me rather unreasonable in Liebig to speak so boldly, as he is wont, respecting physiological phenomena, while making no effort to explain the part performed by electricity in regard to them. Is there not reason to suppose that he has been so much occupied by the analytical department, that he is not sufficiently aware of the difficulty of doing justice to the electro-chemical department of physiology?"

---

At the annual election of the Philadelphia Medical Society, held at their Hall, on Saturday the 6th instant, the following members were chosen officers for the ensuing year, viz:

Prof. R. M. Huston, M. D., *President.*

Benjamin H. Coates, M. D.<br>Prof. Samuel Jackson, M. D. } *Vice Presidents.*

John Wiltbank, M. D., *Treasurer.*

Joseph Warrington, M. D.<br>Isaac Parrish, M. D. } *Corresponding Secretaries.*

John J. Reese, M. D., *Senior Recording Secretary.*

D. Francis Condie, M. D., *Orator.*

Nathan D. Benedict, M. D., *Librarian.*

Aaron D. Chaloner, M. D.<br>Edmund Lang, M. D. } *Curators.*

Job Haines,<br>*Junior Recording Secretary.*

